# Feasibility and Acceptability of a Group-Based Telehealth Stress Management and Resilience Training Intervention for Men with Prostate Cancer on Active Surveillance

**DOI:** 10.3390/jcm15145539

**Published:** 2026-07-15

**Authors:** Nihal E. Mohamed, Jean Claude Noel, Danielle Scharp, Weijia Fu, Himanshu Joshi, Talia Korn, Isabella Johnson, Ashutosh Tewari, Adam Gonzalez

**Affiliations:** 1 Department of Urology, Icahn School of Medicine at Mount Sinai, New York, NY 10029, USA; jeanclaude.noel@mssm.edu (J.C.N.); weijia.fu@mountsinai.org (W.F.); himanshu.joshi@mountsinai.org (H.J.); talia.korn@mountsinai.org (T.K.); ash.tewari@mountsinai.org (A.T.); 2 Division of General Internal Medicine, Icahn School of Medicine at Mount Sinai, New York, NY 10029, USA; danielle.scharp@mountsinai.org; 3 HPV Cancers Alliance, New York, NY 10023, USA; ella@hpvca.org; 4 Department of Psychiatry and Behavioral Health, Renaissance School of Medicine, Stony Brook University, Stony Brook, NY 11794, USA; adam.gonzalez@stonybrookmedicine.edu

**Keywords:** active surveillance, anxiety, prostate cancer, stress management and resilience training, telehealth

## Abstract

**Background/Objectives**: Active surveillance (AS) is the recommended management strategy for localized, early-stage prostate cancer. Despite promising cancer-specific outcomes, up to one-third of men discontinue AS and undergo radical prostatectomy without evidence of disease progression, often because of stress, anxiety, uncertainty, and unmet supportive care needs. Evidence-based psychosocial interventions tailored to men on AS are lacking. We aimed to: (1) adapt the Stress Management and Resilience Training (SMART) program to address the unique psychosocial and supportive care needs of men with prostate cancer on AS (SMART-AS), and (2) evaluate the feasibility and acceptability of SMART-AS. **Methods**: Following the Assessment, Decision, Adaptation, Production, Topical Experts, Integration, Training, Testing (ADAPT-ITT) framework, we adapted SMART for men with prostate cancer on AS (SMART-AS) informed by our prior qualitative study and expert input. Next, we conducted a single-arm pilot feasibility study at one large urban academic medical center. Participants attended eight weekly 90-minute telehealth group SMART-AS sessions. Feasibility and acceptability were evaluated one-week post-intervention. **Results:** Based on our prior qualitative study and expert input, core SMART-AS components included content targeting stress, anxiety, communication, and self-management. In total, 30 participants were enrolled in the pilot feasibility study and completed baseline assessments (mean age = 71 years, standard deviation = 8.3); 26/30 (86.7%) completed six out of eight SMART-AS sessions, and 17/30 (56.7%) completed post-intervention assessments. Nearly all (16/17, 94.1%) reported that they would recommend SMART-AS to others. Most agreed that SMART-AS helped them talk to clinicians (13/17, 76.5%), reduced anxiety (13/17, 76.5%), enhanced coping skills for AS challenges (15/17, 88.2%), and supported self-care (15/17, 88.2%). Nearly two-thirds (11/17, 64.7%) reported SMART-AS helped them continue AS as a management strategy. **Conclusions**: This pilot study provides preliminary evidence supporting the feasibility and acceptability of SMART-AS among men with prostate cancer on AS. Participants reported perceived improvements in anxiety, coping, communication with clinicians, and self-management. Findings support further evaluation in a randomized controlled trial.

## 1. Introduction

Over three million men in the United States have prostate cancer, with 60% of diagnoses occurring in men aged ≥65 years [1]. Prostate cancer is the second most common cancer among men worldwide and remains a major public health challenge due to its high prevalence and long-term survivorship needs [1]. For men with low-risk, localized prostate cancer, treatment options range from active surveillance (AS) to radical prostatectomy [2]. Standard AS protocols involve prostate-specific antigen (PSA) testing every 3–6 months and annual prostate biopsies, with more invasive intervention only upon evidence of disease progression [2]. Notably, cancer-specific survival at 10–15 years for men on AS is approximately 98–99% [3]. Further, AS helps preserve patients’ quality of life by maintaining urinary and sexual function, thereby avoiding adverse outcomes often observed after radical prostatectomy [4]. However, adherence to AS protocols remains a critical challenge [5]. In a multi-national cohort study of over 10,000 men on AS, approximately 13% discontinued AS and opted for radical prostatectomy in the absence of cancer progression [5]. The most common reasons for this decision include stress and anxiety related to living with untreated cancer, difficulty tolerating uncertainty, and perceived lack of control over the disease trajectory [6,7]. Thus, there is a need for targeted interventions to address stress, anxiety, and supportive care needs to help men with prostate cancer on AS continue this management strategy [8].

Stress is a state in which perceived external demands exceed an individual’s available coping resources [9], whereas anxiety is characterized by persistent and excessive worry or apprehension that is difficult to regulate [10]. Stress and anxiety are increasingly recognized as important concerns among men with prostate cancer undergoing active surveillance (AS), yet rigorously tested, evidence-based interventions targeting stress and anxiety in this population are lacking. A small number of studies suggest that cognitive-behavioral therapy and stress management interventions may improve adherence to AS protocols [11,12], while enhancing psychological well-being and health-related quality of life [11,12]. However, these interventions primarily focus on reducing stress and anxiety reduction and do not comprehensively address the broader informational, decisional, and supportive care needs that influence patients’ confidence in continuing AS over time.

The Stress Management and Resilience Training (SMART) program is a telehealth-delivered, manualized eight-week group intervention developed at the Benson-Henry Institute for Mind Body Medicine at Massachusetts General Hospital that integrates cognitive-behavioral and positive psychology techniques, relaxation response elicitation, healthy lifestyle behavior education and goal setting, and peer support [13,14]. The telehealth, group-based format of SMART may be particularly advantageous for men undergoing AS by increasing access to supportive care while fostering peer support and shared coping among men facing similar challenges [15,16,17,18,19]. SMART has been successfully administered via telehealth platforms to address barriers to care and improve adherence to treatment [17,20]. SMART has strong potential to support men with prostate cancer on AS by addressing stress, anxiety, and unmet supportive care needs that may influence AS adherence. Thus, we aimed to: (1) adapt SMART to address the unique psychosocial and supportive care needs of men with prostate cancer on AS (SMART-AS), and (2) evaluate the feasibility and acceptability of SMART-AS.

## 2. Methods

### 2.1. Study Design

This study involved two phases: (1) iterative adaptation of SMART-AS through mapping the evidence from our previously published qualitative study [19] and expert input, and (2) feasibility and acceptability evaluation through a single-arm pilot feasibility study.

#### 2.1.1. Iterative Adaptation

The Assessment, Decision, Adaptation, Production, Topical Experts, Integration, Training, Testing (ADAPT-ITT) framework involves eight phases that span iterative intervention adaptation and pilot testing [21]. A summary of each ADAPT-ITT framework phase objectives and actions are provided in Table 1. We completed the Assessment phase in our prior qualitative study, including men with prostate cancer on AS and clinicians [19]. Key findings used to inform SMART-AS adaptations and are described elsewhere [19] and listed in Supplementary Table S1.

#### 2.1.2. Intervention: SMART-AS

In Table 2, we describe SMART-AS session content and specific adaptations. The finalized SMART-AS intervention consisted of eight weekly 90-minute group videoconference sessions ready for delivery by facilitators using Zoom for Healthcare, a HIPAA-compliant platform. Prior to the first session, participants received a technology orientation call from the research coordinator. Core SMART-AS components included: (1) relaxation response elicitation (e.g., diaphragmatic breathing, body scan, mindful movement, and guided imagery), (2) cognitive restructuring targeting AS-specific negative thoughts, (3) mindfulness and acceptance training for tolerating cancer uncertainty, (4) stress and anxiety awareness and personalized coping plans, (5) social support mobilization and patient–clinician communication skills, and (6) healthy lifestyle behavior education and goal setting, including sleep hygiene, physical activity, social support, and nutrition with prostate cancer-specific evidence. Between sessions, participants were encouraged to engage in daily skills practice using practice logs, including eliciting the relaxation response for 5–20 min per day, setting weekly healthy lifestyle behavior goals, engaging in adaptive perspective taking, behavioral activations, and recording daily appreciations. Participants were also encouraged to monitor the frequency and distress ratings of any psychological or physical health symptoms.

### 2.2. Pilot Feasibility Study

#### 2.2.1. Study Setting, Participants, and Recruitment

This study was conducted at the Department of Urology at the Icahn School of Medicine at Mount Sinai (ISMMS) in New York City. Eligibility criteria were men who had histologically confirmed prostate adenocarcinoma, were actively enrolled in an AS protocol, receiving ongoing urological care at ISMMS, had an internet-enabled device with video capability, spoke English, and could provide written informed consent. Exclusion criteria were men who progressed from AS management to active treatment, had significant cognitive impairment, unstable psychiatric conditions, or the inability to commit to attending telehealth SMART sessions. Following pilot feasibility study recommendations [22], our target enrollment was 20–30 participants. Potentially eligible participants were identified through systematic screening of the ISMMS Data Warehouse using ICD-10 code C61 (i.e., malignant neoplasm of the prostate) combined with documentation in the electronic health record (EHR) of AS protocol enrollment. Recruitment strategies included introduction of the study to patients in clinic by treating urologists, telephone contact, and mail outreach.

#### 2.2.2. Informed Consent

Written informed consent was obtained from all participants. For participants recruited remotely, the research coordinator reviewed the full consent document verbally via telephone or videoconference, with signed documents returned via secure email or mail. The consent document covered the study purpose and procedures, voluntary participation and right to withdraw without impact on clinical care, risks and benefits, confidentiality protections, compensation (i.e., $30 gift card), and EHR data access and videoconference recording permissions. The study was approved by the ISMMS Institutional Review Board (Protocol #STUDY-2300337, approved on 12 December 2023). This study was reported in accordance with the Consolidated Standards of Reporting Trials (CONSORT) extension guidance for pilot and feasibility studies [23].

#### 2.2.3. Data Collection and Measures

All data were collected and managed using Research Electronic Data Capture (REDCap) [24]. Baseline assessments included sociodemographic and clinical characteristics, social determinants of health screening [25], and psychological distress measures including the Hospital Anxiety and Depression Scale [26], Generalized Anxiety Disorder-7 [27], Patient Health Questionnaire-9 [28], Perceived Stress Scale-10 [29], and the Memorial Anxiety Scale for Prostate Cancer [30]. Psychological distress measures were also assessed one-week post-intervention and corresponding outcome analyses will be reported separately. Additional one-week post-intervention assessments included an investigator designed 13-item feasibility and acceptability questionnaire directly derived from the unmet needs identified in our prior qualitative study to assess perceived utility for healthcare planning, AS-specific benefits, self-care support, coping skill development, recommendation endorsement, material engagement, and application to clinical encounters [19]. Responses for each item ranged from 1 (strongly agree) to 4 (strongly disagree), with higher scores indicating greater dissatisfaction with the intervention. Each item was analyzed separately to evaluate specific aspects of intervention acceptability (e.g., perceived helpfulness for improving communication, knowledge, and burden of care); therefore, an overall summary score was not calculated. For descriptive reporting, responses of strongly agree and agree were considered favorable indicators of acceptability. This investigator-developed questionnaire has been tested in our prior study [19]. Feasibility was defined as ≥70% of participants attending at least six of the eight SMART-AS sessions [31]. Acceptability was defined as ≥70% of participants reporting positive evaluations of SMART-AS [32], including its perceived helpfulness in improving self-care, psychological adjustment, and patient-clinician communication. Acceptability outcomes are based on data from participants who completed post-intervention assessments.

To capture qualitative process data to contextualize quantitative findings, a member of our research team with expertise in psychodynamic group processes (JCN) served as a session observer. Observer notes were structured using a standardized process observation guide developed a priori based on key intervention components and our prior qualitative findings [19]. The process observation guide was designed to capture session attendance and participant engagement (e.g., video-on rates, chat participation, verbal contributions, and body language), fidelity to session content and timing, facilitator adherence to SMART-AS adaptations and AS-specific examples, participant responses to specific AS-adapted content, technical difficulties, and unsolicited participant comments to derive emerging themes. Observer notes were recorded in real-time during sessions and expanded within 24 h of each session. Observer notes were reviewed by the principal investigator (NEM) weekly to identify content areas requiring reinforcement or modification, emergent participant needs not captured by the quantitative measures, and fidelity concerns requiring facilitator feedback.

#### 2.2.4. Statistical Analysis

Following recommendations for pilot studies [22], feasibility and acceptability analyses focused on descriptive statistics and parameter estimation rather than formal hypothesis testing. We described continuous variables using means and standard deviations and categorical variables using frequencies and proportions. Given that acceptability was assessed only after participants completed the intervention, analyses were conducted using available-case data from participants who completed the post-intervention assessment [33].

## 3. Results

### 3.1. Recruitment and Enrollment

**Figure 1:**
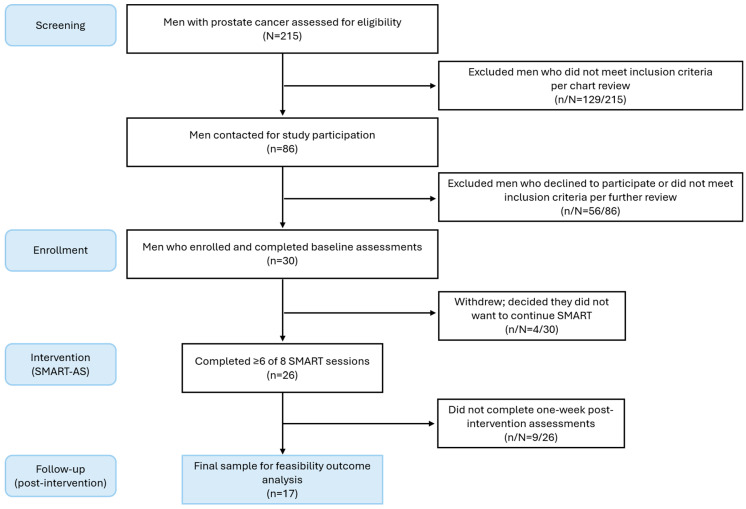
Study flow diagram.

The study flow diagram is provided in Figure 1. Between 18 December 2023 and 8 December 2025, 215 prostate cancer patients were assessed for eligibility from the department of urology, and 86 potentially eligible men were contacted. In total, 56/86 (65.1%) did not meet the inclusion criteria or declined to participate, and 30/86 (35.0%) consented and enrolled, meeting our target recruitment goal. The primary reason for declining participation included scheduling conflicts. Participants were organized into five groups of 3-8participants.

### 3.2. Sample Characteristics

In total, 30 participants completed baseline assessments (100% data completeness). The sample had a mean age of 71 years (standard deviation = 8.3). Most were married (22/30, 73.3%) and reported their race as white (26/30, 86.7%). Approximately half had graduate degrees (14/30, 46.7%) and half were retired or semi-retired (15/30, 50.0%). Half (15/30, 50%) were diagnosed with prostate cancer within the past 5 years. The most frequently discussed treatment options included surgery (11/30, 36.7%), watchful waiting (9/30, 30.0%), and external beam radiation (6/30, 20.0%). Participants reported generally favorable overall health, with 12/30 (40.0%) rating their health as “very good” and 7/30 (23.3%) rating their health as “excellent.” Additional sample characteristics are provided in Table 3.

### 3.3. Observer Notes and Process Observations

A summary of the observer notes is presented in Table 4. Structured observer notes across all sessions documented several important process observations that contextualize the quantitative data. Key themes included the value of peer support and normalization, benefits of addressing PSA-related stress and anxiety, importance of facilitating open emotional expression, utility of communication skills to engage with clinicians, relevance of guidance on nutrition and physical activity, and the desire for family or partner involvement.

### 3.4. Outcomes

Among 30 enrolled participants, 26/30 (86.7%, 95% CI: 69.3–96.2) attended at least six out of eight SMART-AS sessions, meeting our pre-specified feasibility benchmark. Primary reasons for missed sessions included scheduled medical appointments, temporary illness, family obligations, and occasional technology difficulties, although technology barriers were minimal. All 30 participants successfully connected to videoconference sessions after the initial orientation call. Four participants withdrew because they decided that they did not want to continue the intervention due to reluctance to discuss sensitive topics in a group format. In total, 17/30 participants (56.7%, 95% CI: 37.8–74.5) completed one-week post-intervention assessments. Among the 17 participants who completed post-intervention assessments, overall acceptability was high. Nearly all participants (16/17, 94.1%) reported they would recommend the intervention to others. High endorsement was also observed for self-care and coping, with 15/17 (88.2%) indicating the program helped them take better care of themselves and cope with challenges related to AS and 11/17 (64.7%) reporting improvements in anxiety symptom management.

Most participants (13/17, 76.5%) agreed that SMART-AS improved communication with clinicians, increased knowledge, enhanced confidence, and clarified healthcare priorities. Approximately two-thirds (11/17, 64.7%) reported increased knowledge about AS and support for continuing AS. Engagement with intervention materials outside of SMART-AS sessions was moderate, with 8/17 (47.1%) reporting ≤5 h of review and 5/17 (29.4%) reporting ≥6 h. Approximately half (8/17, 47.1%) reported applying knowledge gained during clinical consultations (Table 5).

## 4. Discussion

This study supports the feasibility and acceptability of SMART-AS, a telehealth-delivered, group-based stress management intervention adapted for men with prostate cancer on AS. SMART-AS was developed through a rigorous formative process grounded in our team’s previous qualitative research [19] and guided by the ADAPT-ITT framework [21]. Most participants reported perceived improvement in stress and anxiety, engagement with their AS care, coping skills while on AS, and self-care strategies that they perceived as supporting their ability to continue AS, addressing critical unmet needs among this population.

Our formative qualitative work [19] provides a direct lineage from patient and clinician insight to SMART-AS content, which may have contributed to the observed feasibility and acceptability outcomes by ensuring that the intervention reflected perceived concerns and priorities of men on AS. Most participants reported that the program helped them manage stress and anxiety related to AS and nearly all indicated that it helped them better cope with the challenges of living with untreated prostate cancer and ongoing surveillance. Importantly, the need for continuity and improved communication in follow-up care was also addressed, as SMART-AS encouraged participants to discuss healthcare decisions and concerns with their clinicians. Taken together, these findings suggest that the adapted program was perceived to address several of the supportive care needs identified in our prior qualitative work, including uncertainty management, emotional support, and communication with clinicians [19].

Connecting with others is an important priority among men on AS [19]. The group-based format of SMART-AS addresses this need by facilitating peer connections and group discussions about experiences and challenges in similar contexts. Observer notes documented that peer normalization emerged as one of the most therapeutically meaningful elements across SMART-AS sessions, with participants repeatedly expressing relief at discovering shared experiences. This is consistent with prior research demonstrating that peer support can reduce isolation and validates the emotional burden of living with untreated cancer [34]. Further, participants became advocates for the peer support model, suggesting that they perceived hearing from someone who has “gone through the disease” as valuable and potentially supportive of selecting or continuing AS.

Inadequate emotional and informational support is likely a major determinant in the decision to continue AS, even among those who were initially interested in AS as a management strategy [19]. Most participants reported that SMART-AS helped them continue AS as a management strategy, suggesting that participants perceived the program as providing psychosocial support for remaining on AS in a population that was already committed to AS but lacked sufficient psychosocial scaffolding to sustain that commitment over time. While prior studies have largely focused on the initial decision to choose AS over treatment [35], our findings suggest that participants perceived ongoing psychosocial support and adaptive coping skills as important for remaining on AS. This distinction between willingness to continue AS and adequate support to do so is precisely the gap our formative qualitative work identified and SMART-AS was designed to address.

PSA-related concerns were identified as the dominant ongoing source of stress and anxiety for men on AS. This aligns with evidence that PSA testing triggers a recurring cycle of stress and anxiety, which peaks in the days before and after results [36]. Specific SMART-AS adaptations addressed this, particularly the mindful awareness exercise for anticipatory anxiety and cognitive restructuring for negative thoughts about the results. These strategies were among the intervention components with the highest levels of participant engagement. Additionally, SMART-AS provided portable coping tools, such as brief relaxation response elicitation practices for real-time use. While prior interventions have addressed general cancer-related anxiety, few have targeted temporal stress and anxiety, such as that surrounding PSA monitoring visits, representing a novel contribution of SMART-AS in this context [37].

Men on AS may lack skills to effectively express concerns, ask questions, or participate actively in shared decision-making [19]. This is consistent with broader evidence that patients’ lack of knowledge and limited communication skills are key barriers to active participation in cancer care decisions [38]. Nearly half of participants reported applying knowledge gained from SMART-AS in clinical consultations, suggesting that participants perceived the communication skills training as useful in supporting discussions with their clinicians. While most communication interventions in oncology have focused on training clinicians, few have equipped patients with skills to advocate for their needs during consultations, particularly in the AS context where ongoing shared decision-making is essential [39,40,41].

Men on AS may have family involvement in AS care. While this may be perceived as a resource for support, family involvement may compound patient anxiety [19]. This is consistent with evidence that prostate cancer is increasingly recognized as a “couple’s disease,” with partners experiencing significant psychological distress that correlates with patient distress [42]. While family involvement during the decision-making process is suggested as part of psychosocial support for men on AS [42], careful consideration regarding the timing and duration of such involvement is warranted. Although some participants identified their family member’s absence from the program as a format they preferred, others requested their inclusion in future iterations. Notably, the social support diagram and empathy communication exercises delivered in the current SMART-AS surfaced partner-related concerns from patients’ perspectives that had not previously been verbalized. While emerging evidence from dyadic interventions in oncology suggests that partner-based approaches can improve communication and reduce cancer-related distress for partners, effects on patient anxiety are more variable and depend on intervention design, including duration and content [43]. Dyadic design may be beneficial for some SMART-AS sessions, while the current patient-only format may be better for more sensitive or individualized topics. These changes should be considered for future iterations of SMART-AS and tested by obtaining data from both patients and partners.

Nutritional and physical activity guidance are necessary components of AS care [19]. This is supported by growing evidence that healthy dietary patterns, particularly Mediterranean and plant-based diets, and regular physical activity are associated with reduced prostate cancer progression and improved survival outcomes [44]. Additionally, guidelines recommend incorporating diet and exercise counseling into prostate cancer survivorship care [35]. Participants actively engaged with SMART-AS sessions on healthy eating and physical activity, with participants actively sharing their lifestyle modification strategies with peers. Most participants agreed that SMART-AS helped them take care of themselves in terms of their overall physical health and well-being, suggesting that they perceived this content as relevant and empowering. Lifestyle interventions may have the potential to influence prostate cancer-specific anxiety, fear of cancer progression, and self-esteem, suggesting that focusing on nutrition and physical activity may serve as a health-promoting and anxiety-reducing strategy for men on AS [45].

Community-based participatory research approaches should be considered in future SMART-AS studies, particularly partnerships with community organizations and targeted recruitment strategies to engage men from diverse racial, ethnic, educational, and socioeconomic backgrounds. Community-based dissemination and educational outreach efforts may also help build trust, increase awareness of AS and supportive care resources, and facilitate engagement among men who may otherwise be reluctant to participate in research or supportive interventions [46]. These efforts may be particularly valuable when considering involving spouses or partners, who often play an important role in encouraging men to engage in healthcare decision-making and supportive care [47]. Notably, cultural and family dynamics have been identified as facilitators of prostate cancer care engagement among racially and ethnically minoritized populations, suggesting that community-based outreach strategies that leverage these strengths may be effective. Additionally, while the telehealth format of SMART-AS may increase access, it paradoxically exacerbates disparities for men with limited digital literacy or internet access [48]. Prior research has documented racial and ethnic differences in being offered or accepting telehealth for cancer care, with technology-related concerns serving as key barriers to engagement [49]. These considerations underscore the importance of integrating culturally responsive, community-engaged, and digitally inclusive strategies in future studies and future implementation of SMART-AS.

### 4.1. Strengths and Limitations

Key strengths of this study include its grounding in rigorous formative qualitative research, which enhanced the relevance of SMART-AS. Our intervention targeted clearly defined and consistently identified unmet needs from patient and clinician perspectives, strengthening its clinical relevance. The adaptation process was guided by the ADAPT-ITT framework [21], with all eight phases documented to ensure transparency and reproducibility. The group-based telehealth delivery model directly addressed peer support needs and potential access barriers identified in our qualitative work, while supporting feasibility and future scalability. Structured observer notes provided valuable process-level data that helped contextualize quantitative findings and interpret participant engagement and response.

There are also some limitations. This was a small, single-arm pilot study without a control group or long-term follow up, which precludes inferences regarding intervention effects. Participants were recruited from a single academic medical center and self-selected into the study, potentially resulting in a sample that was more motivated to participate in a behavioral intervention than the broader population of men on AS. Retention from baseline to post-intervention was moderate, and loss to follow-up may have introduced response bias. Interpretation of acceptability findings should consider that only 17 of 30 participants completed the post-intervention assessment. Because acceptability measures were collected only after intervention completion, estimates reflect the experiences of completers and may overestimate overall acceptability if participants with less favorable experiences were more likely to discontinue. The sample was comprised of participants who reported their race as white, who were highly educated, and of relatively high socioeconomic status, limiting generalizability to more diverse populations and clinical settings. This study did not include longer-term follow-up, precluding assessment of sustained effects on AS adherence or quality of life. Outcomes were assessed with self-report measures supplemented by objective observer notes, without clinician-reported outcomes or objective measures of physiological anxiety responses. Additionally, acceptability was assessed using an investigator-developed questionnaire designed to assess intervention-specific aspects of acceptability. Accordingly, individual items were summarized rather than combined into an overall score. Although observer notes provided valuable insight in this study, these observations reflected the perspective of a single observer and may not fully capture variability in participant experiences.

### 4.2. Future Research Directions

Building on the evidence from both the qualitative study and this pilot feasibility study, future research should include a randomized controlled trial comparing SMART-AS to usual care with adequate power to detect effects on anxiety, quality of life, and AS adherence over time. Future studies should also prioritize intentional recruitment of racially, ethnically, and socioeconomically diverse participants through community-engaged approaches and partnerships with trusted community representatives. This may improve participation among men who may be reluctant to engage in group-based supportive interventions related to prostate cancer. Additionally, more work is needed to better understand how social determinants of health shape anxiety, coping, and engagement with supportive care interventions among men on AS. Dyadic versions of SMART-AS incorporating spouses or partners should also be explored. Additional priorities include examining who may benefit most from components of SMART-AS based on baseline anxiety, time on AS, coping styles, and social context, evaluating cost-effectiveness and integration into routine AS protocols, and investigating changes in uncertainty tolerance, patient-clinician communication, and anxiety physiology as mediators of SMART-AS effects.

## 5. Conclusions

This pilot study supports the feasibility and acceptability of SMART-AS among men with prostate cancer on AS. Participants perceived that SMART-AS addressed important supportive care needs related to stress, anxiety, coping, communication with clinicians, and self-management. These findings provide preliminary support for the use of a group-based, telehealth-delivered psychosocial intervention in this population and justify further evaluation in a randomized controlled trial. Future research should evaluate the effectiveness of SMART-AS in a larger, more diverse population, examine long-term outcomes including anxiety, quality of life, and adherence to AS, and explore future implementation within routine clinical care.

## Figures and Tables

**Table 1:** Application of the ADAPT-ITT Framework to Adapt SMART for Men with Prostate Cancer on Active Surveillance, Informed by Mohamed et al. (2018) [19].

Phase	Objective	Action Taken	Qualitative Finding Applied
Iterative Adaptation and Pre-Testing			
Assessment	Identify population needs and preferences	Targeted review of published qualitative literature; application of findings from Mohamed et al. (2018) [19]: which achieved thematic saturation through interviews with 28 AS patients and three clinicians at ISMMS	Six unmet need domains identified: informational needs, anxiety/stress management, family caregiver involvement, follow-up reminders, lifestyle changes, peer support
Decision	Select evidence-based intervention for adaptation	SMART selected based on prior efficacy for anxiety, stress, and lifestyle changes; group format addresses peer support need; telehealth delivery addresses geographic barriers to appointments	Patients reported travel burden to appointments as a stressor; 79% wanted peer connection; 82% reported emotional distress requiring structured intervention
Adaptation	Modify intervention content and format	All eight sessions adapted to incorporate AS-specific stressors, PSA anxiety management, masculine identity and sexual functioning concerns, clinician communication skills, and family involvement	Direct mapping of qualitative themes to session content, e.g., PSA anxiety → Session 3 mindful awareness; peer support → group format
Production	Develop adapted materials	Created AS-specific facilitator manual, participant workbook, audio RR recordings, and session slides with AS examples and terminology	Patients requested brochures, care plans, and accessible formats; older adults preferred paper + digital hybrid materials
Topical Experts	Solicit expert feedback	Review by two urologists, two clinical psychologists, and one patient advocate	Clinician interviews highlighted need for universal language about AS; experts ensured AS protocol descriptions were clinically accurate
Integration	Refine intervention based on expert feedback	Incorporated expert recommendations: reframing AS as “active monitoring not passive waiting”; adding polypharmacy and comorbidity content for older adults; strengthening clinician communication module	Clinicians noted patients limited understanding of the AS protocol; patients wanted clearer information about follow-up tests and schedules
Training	Prepare facilitators for delivery	Facilitator (AG; licensed clinical psychologist, 10+ years health psychology/group therapy experience) trained on SMART program, AS-specific concerns, and telehealth delivery best practices + trained observer with expertise in psychodynamic group processes (JCN)	Clinicians identified lack of mental health resources; facilitator training included AS clinical context and psychosocial challenges specific to prostate cancer surveillance protocol
Pilot Feasibility Study			
Testing	Pilot adapted intervention	Tested SMART-AS in present pilot study; evaluated feasibility and acceptability (results reported herein)	Full formative cycle complete; pilot tests all adaptations in the target population prior to larger trial

**Table 2:** SMART Program Session Content and Adaptations for Men with Prostate Cancer on Active Surveillance (SMART-AS), Grounded in Formative Qualitative Findings (Mohamed et al., 2018) [19].

Session	Core SMART Module	Key SMART-AS Adaptations
S1	Introduction to Mind–Body Medicine	Introduced AS as active monitoring rather than passive observation; addressed AS-specific education (PSA schedule, biopsy expectations) and stressors including PSA anxiety and surveillance fatigue.
S2	Relaxation Response	Applied relaxation techniques to cancer-related sleep disturbance and anticipatory anxiety, particularly around PSA testing and result notification.
S3	Stress Awareness	Focused on identifying AS-specific stress triggers (PSA tests, biopsies, uncertainty), strengthening social support, and integrating prostate-healthy nutrition education.
S4	Mending Mind & Body	Addressed negative thoughts related to AS (e.g., fear of progression), coping with surveillance stress, and concerns related to sexual wellness and functioning.
S5	Creating an Adaptive Perspective	Promoted cognitive reframing of AS, acceptance of uncertainty, coping with PSA fluctuations, and lifestyle modification through healthy diet practices.
S6	Promoting Positivity	Reinforced optimism, physical activity, and adaptive routines to support resilience, improve quality of life, and reduce fear of progression.
S7	Healing States of Mind	Emphasized self-compassion, acceptance, root fears, emotional expression, communication with partners, and engagement with the clinical care team.
S8	Humor, Empathy & Staying Resilient	Consolidated coping skills, relapse prevention, adherence planning, and long-term resilience strategies to support sustained AS engagement.

Note. Original SMART content based on the Benson-Henry Institute for Mind Body Medicine Stress Management and Resiliency Training program. AS = Active Surveillance; PSA = Prostate-Specific Antigen.

**Table 3:** Sociodemographic and Clinical Characteristics of Men on Active Surveillance Who Enrolled in the SMART-AS Pilot Study and Completed Baseline Assessments (N = 30).

Characteristic	n (%)
Age, mean (SD)	71.0 (8.3)
Education level	
Vocational/technical school	1 (3.3)
Some college	4 (13.3)
Bachelor’s degree	11 (36.7)
Graduate degree	14 (46.7)
Marital status	
Never married	3 (10.0)
Married	22 (73.3)
Divorced or separated	3 (10.0)
Widowed	2 (6.7)
Race or ethnicity	
White	26 (86.7)
Hispanic	1 (3.3)
Asian	1 (3.3)
Other	2 (6.7)
Employment status	
Employed	14 (46.7)
Unemployed	1 (3.3)
Retired or semi-retired	15 (50.0)
Insurance type	
Medicare only	4 (13.3)
Medicare + supplemental	11 (36.7)
Private	14 (46.7)
Missing	1 (3.3)
Years since diagnosis	
<5 years	15 (50.0)
≥5 years	12 (40.0)
Does not know/Missing	3 (10.0)
Treatment options discussed	
Surgery	11 (36.7)
External beam radiation	6 (20.0)
Watchful waiting	9 (30.0)
Brachytherapy	2 (6.7)
Hormones	1 (3.3)
Overall self-rated health	
Excellent	7 (23.3)
Very good	12 (40.0)
Good	5 (16.7)
Fair	4 (13.3)
Poor	1 (3.3)
Missing	1 (3.3)
Smoking status	
Current smoker	0 (0)
Former smoker	8 (26.7)
Never smoker	22 (73.3)
Social determinants of health	
Food insecurity	2 (6.7)
Stable housing	29 (96.7)
Unable to access utilities	2 (6.7)
Transportation barriers	2 (6.7)
Physically and emotionally safe	28 (93.3)
Emotionally abused by partner	2 (6.7)

Note. “Other” race or ethnicity includes Afro-Caribbean and African. “Treatment options discussed” reflects a multi-select survey item. Social determinants of health were assessed with the Centers for Medicare and Medicaid Services Accountable Health Communities screening items.

**Table 4:** Summary of SMART-AS Session Observer Notes.

Domain	Process Observations
Participant Engagement	Engagement varied across cohorts and over time. Some participants were highly interactive from early sessions, whereas others initially demonstrated limited verbal participation but became more engaged as sessions progressed. Greater participation was often observed during structured experiential exercises compared with open-ended discussions.
Group Dynamics	Group cohesion generally increased over time, with many participants reporting greater comfort sharing personal experiences as trust developed. Peer support emerged around shared active surveillance (AS) experiences, including PSA testing, biopsies, and uncertainty related to disease monitoring. Smaller cohorts allowed for more individualized discussion but were more sensitive to attendance variability.
Attendance and Retention Challenges	Attendance variability was observed across cohorts, with scheduling conflicts, competing work responsibilities, personal stressors, health-related concerns, and technical barriers contributing to missed sessions or attrition. In smaller cohorts, inconsistent attendance affected continuity of group interaction.
Common Sources of Stress	Frequently reported stressors included uncertainty related to AS, PSA testing and result anticipation, biopsy procedures, fear of disease progression, work and financial stress, family responsibilities, and broader societal stressors. These stressors often interacted with participants’ cancer-related concerns.
Implementation Challenges	Facilitators observed several implementation challenges, including maintaining group boundaries, accommodating variable comfort levels with emotional disclosure, addressing technical difficulties during virtual participation, and balancing educational content with experiential exercises. Some participants preferred practical skills-based content over reflective exercises.
Observed Intervention Use	Participants reported applying intervention strategies both to cancer-related and non-cancer-related stressors. Commonly used skills included relaxation response exercises, brief “mini” practices, mindfulness techniques, cognitive reframing, and stress monitoring. Participants frequently described using these tools during periods of acute stress or uncertainty.
Observed Perceived Benefits	Process observations noted increased stress awareness, emotional regulation, coping confidence, sleep, and communication with family and clinicians. Participants also demonstrated increased awareness of personal stress triggers and greater use of adaptive coping strategies over time. Peer normalization and shared discussion of AS experiences appeared to reduce feelings of isolation.

**Table 5:** Feasibility and Acceptability of SMART-AS One Week Post-Intervention (n/N = 17/30).

Item	Agree n/N (%)	Disagree n/N (%)	Do Not Know n/N (%)
Would you recommend this program to other patients like you?	16/17 (94.1)	1/17 (5.9)	0/17 (0)
This program helped me take care of myself	15/17 (88.2)	1/17 (5.9)	1/17 (5.9)
This program taught me how to cope with the challenges of being on active surveillance	15/17 (88.2)	0/17 (0)	2/17 (11.8)
This program helped me talk to doctors/nurses about my healthcare planning	13/17 (76.5)	2/17 (11.8)	2/17 (11.8)
This program increased my knowledge about my healthcare planning	13/17 (76.5)	2/17 (11.8)	2/17 (11.8)
This program made me feel less anxious or upset about my healthcare planning	13/17 (76.5)	4/17 (23.5)	0/17 (0)
This program made me feel more confident about my healthcare planning	13/17 (76.5)	1/17 (5.9)	3/17 (17.6)
This program sorted out what’s important to me for dealing with my healthcare planning	13/17 (76.5)	2/17 (11.8)	2/17 (11.8)
This program increased my knowledge about active surveillance	11/17 (64.7)	4/17 (23.5)	2/17 (11.8)
This program helped me continue being on active surveillance management	11/17 (64.7)	4/17 (23.5)	2/17 (11.8)
This program helped me manage/control my symptoms	11/17 (64.7)	3/17 (17.6)	3/17 (17.6)
Hours reviewing SMART-AS materials None ≤5 6–10 ≥10 Missing	2/17 (11.8) 8/17 (47.1) 2/17 (11.8) 3/17 (17.6) 2/17 (11.8)		
Used SMART-AS knowledge in clinical consultations Yes No Missing	8/17 (47.1) 8/17 (47.1) 1/17 (5.9)		

## Data Availability

The data presented in this study are available on request from the corresponding author due to privacy and ethical restrictions related to participant confidentiality.

## Supplementary Materials

The following supporting information can be downloaded at: https://www.mdpi.com/article/10.3390/jcm15145539/s1, Table S1: Summary of Key Qualitative Findings from Men with Prostate Cancer on AS and Their Application to the Proposed SMART-AS Adaptations.

## Abbreviations

The following abbreviations are used in this manuscript:

AS	Active surveillance
SMART	Stress Management and Resilience Training
ADAPT-ITT	Assessment, Decision, Adaptation, Production, Topical Experts, Integration, Training, Testing
PSA	Prostate-specific antigen
SMART-AS	Stress Management and Resilience Training-Active Surveillance
ISMMS	Icahn School of Medicine at Mount Sinai
HIPAA	Health Insurance Portability and Accountability Act
