# Supplementary material for: Feasibility and Acceptability of a Group-Based Telehealth Stress Management and Resilience Training Intervention for Men with Prostate Cancer on Active Surveillance
Source: J Clin Med. 2026 Jul 15;15(14):5539. doi: 10.3390/jcm15145539 (PMC13412582; doi:10.3390/jcm15145539)
Supplement: Supplementary file 1 [file jcm-15-05539-s001.zip › jcm-4398467-supplementary.pdf]

Supplementary Table S1. Summary of Key Qualitative Findings from Men with Prostate Cancer on AS and Their Application to the Proposed SMART-AS Adaptations

Domain	Key Findings	Representative Patient Quotes	Proposed SMART-AS Adaptations
Unmet informational needs at diagnosis	• Independently searched for information about AS and treatment options due to lack of information provided by clinicians at diagnosis	"I knew nothing about AS, I just wanted to understand what it was"	Session 1: AS education and prostate cancer frequently asked questions
Worry and anxiety during AS	• Concerns about not removing a potentially progressive tumor<br>• Emotional distress including fear of cancer progression and PSA-related anxiety	"Cancer is constantly in my mind. I think that if I had decided to have surgery I would have put that out of my mind"	Sessions 1–4: RR elicitation practices; cognitive restructuring targeting cancer-specific thoughts; mindful awareness for PSA testing related anxiety
Difficulties and challenges during the AS protocol	• Uncertainties about of living with a tumor, side effects from biopsies and MRIs, and difficulty keeping up with frequent appointments	"I'm kind of in the midst of getting a high PSA score and that creates anxiety" —	Session 5: Tolerating uncertainty; adaptive perspectives on surveillance; Stop, Breathe, Reflect, Choose (SBRC) tool for acute anxiety around receiving PSA results
Physician trust and shared decision-making	• Followed clinician recommendations based on trust in clinician expertise	"I have a lot of faith in him. I was gonna go along with it"	Sessions 3 & 7: Patient-clinician communication skills; role-play of expressing concerns to clinicians, social support diagram including clinicians
Partner and family involvement	• Caregiver influence on care decisions<br>• Had family involved in AS decision; partner/spouse anxiety about recurrence was significant	"My wife was involved with me the entire time, she went with me in all the appointments"	Session 3: Social support diagram adapted to AS-specific networks; Session 7: empathy exercise extended to include partner distress and dyadic communication skills

Desire for peer support	• Wanted to connect with other AS patients; valued hearing from men who had been on AS successfully	"If you know somebody else who has gone through the disease talk to them about what they went through"	Group-based format of SMART-AS directly to address peer support need; all 8 sessions structured as group-baed with shared learning and normalization
Lifestyle change needs	• Need for additional health services including dietary guidance, physical activity program, and reminders for follow-up appointments	"Find a good doctor, stay in good physical shape, don't gain weight, eat properly…keep a close eye on your condition"	Sessions 5 & 6: Healthy eating module with prostate cancer nutritional guidance; physical activity recommendations tailored for men with prostate cancer
Clinician-identified challenges	• PSA fluctuation-driven anxiety; anxiety due to the absence of universal AS protocol; need for mental health referral pathways	"That's the biggest challenge we face for active surveillance patients, is how do we treat their anxiety"	Facilitator training incorporated clinician-identified challenges; Session 1: education about normalizing AS protocol variation; structured RR tools provided as anxiety management strategies

Note. Qualitative data from Mohamed et al. (2018). RR = Relaxation Response; PSA = Prostate-Specific Antigen; SBRC = Stop, Breathe, Reflect, Choose; AS = Active Surveillance.
